# The Effect of ZrO_2_ Addition and Thermal Treatment on the Microstructure and Mechanical Properties of Aluminum Metal Matrix Composites (AMMCs)

**DOI:** 10.3390/ma18194507

**Published:** 2025-09-28

**Authors:** Isai Rosales-Cadena, Reyna Anahi Falcon-Castrejon, Rene Guardian-Tapia, Jose Luis Roman-Zubillaga, Sergio Ruben Gonzaga-Segura, Lazaro Abdiel Falcon-Franco, Victor Hugo Martinez-Landeros, Rumualdo Servin

**Affiliations:** 1 Center for Research in Engineering and Applied Sciences CIICAp, Autonomous University of Morelos State, Cuernavaca 62209, Morelos, Mexico; reyna.falconcas@uaem.edu.mx (R.A.F.-C.); rene.guardiant@uaem.edu.mx (R.G.-T.); jlroman@uaem.mx (J.L.R.-Z.); 2 Institute of Physical Sciences, ICF-UNAM, Av Univ. 1001 Col. Chamilpa, Cuernavaca 62210, Morelos, Mexico; sergio.gonzaga@icf.unam.mx; 3 Faculty of Metallurgy, Autonomous University of Coahuila, Highway 57 km 5, Monclova 25710, Coahuila, Mexico; lazarofalcon@uadec.edu.mx (L.A.F.-F.); vmartinezlanderos@uadec.edu.mx (V.H.M.-L.); 4 Faculty of Mechanical Electric Engineering, Autonomous University of Coahuila, Barranquilla S/N, Monclova 25280, Coahuila, Mexico; rumualdo.servin@uadec.edu.mx

**Keywords:** AMMCs, particle reinforcement, heat treatment, hardness, wear

## Abstract

Aluminum metal matrix composites (AMMCs) were obtained using the stir-casting method, adding 0.15, 0.25, and 0.50 in vol.% of ZrO_2_. Microstructural observations made using scanning electron microscopy (SEM) indicated that oxide addition modified grain size. X-ray diffraction analyses revealed that mainly ZrAl_3_ and Al_2_O_3_ phases had formed. Hardness evaluation indicated a maximum value of 63 HV for the zirconia-reinforced samples, representing an increase of approximately 70% compared with pure aluminum. This hardness increase was mainly attributed to the zirconia distribution in the aluminum matrix promoting lattice distortion, which promoted the inhibition of dislocation mobility. Wear tests indicated that the samples with 0.50 vol.% of ZrO_2_ added presented the lowest wear rate because of the hardness they acquired. The results are discussed considering composite strengthening due to ZrO_2_ addition and the thermal treatment applied (cooling rate).

## 1. Introduction

Aluminum alloys can acquire good properties, such as improved mechanical and physical properties, when other elements, which offer their own characteristics, are added to them [1]. These improved alloys are needed in structural applications in aeronautics, the automotive industry, transportation, etc. In the last few decades, aluminum alloys with reinforcing particles have formed a new class of materials called aluminum metal matrix composites (AMMCs), which have been found to have excellent properties for several applications in different industries [1,2,3]. Aluminum-based composites combine the properties of metal alloys and reinforcements, giving them improved properties, such as high stiffness and tensile strength, superior wear resistance, a controlled thermal expansion coefficient, and fatigue resistance [2,3], enabling the creation of materials for specific applications [4,5,6]. One of the main advantages of producing metallic metal composites (MMCs) is the possibility of obtaining materials with improved characteristics, such as high hardness and elevated melting points. Among the methods used to produce reinforced composites, the most efficient are mechanical alloying [7,8], infiltration, hot pressing, and powder metallurgy [9,10,11,12,13]. Melt stirring can also be considered an effective alternative for producing MMCs since it can be easily applied and provides reproducible results [14,15,16]. Therefore, in this study, we used the melt-stirring method to obtain an aluminum metal matrix composite (AMMC) supplemented with metal oxides such as ZrO_2_, which has excellent tensile resistance, relatively good fracture toughness, and a low density [17], making it an ideal candidate for use as reinforcement. Wear and hardness evaluations of soft materials like aluminum alloys, specifically in AMMCs, constitute a topic that requires high concentration during the development of tests mainly because of these composites’ relatively low hardness, which plays an important role in the mechanical behavior of the alloy. Reddy et al. [18] investigated the influence of ZrO_2_ nanoparticles on the mechanical and tribological properties of the AA7075 alloy supplemented with ZrO_2_ (zirconium dioxide) particles in different contents to create a composite using the stir-casting technique. Boppana et al. [19] studied the effects of the addition of nano-graphene and ZrO_2_ from mechanical and microstructural perspectives to produce Al 6061 metal matrix composites. The influence of ZrO_2_ on the tribological and mechanical properties of a reinforced composite made using the stir-casting technique has also been studied recently [20]. Several authors have investigated the Al-Zr system from a thermodynamic perspective [21,22]. Nevertheless, there is still no specific information on the mechanical characteristics of Al-ZrO_2_ composites. Thus, the novelty of this work lies in the amount of reinforcement added to the composite as well as in the way we added the ZrO_2_ particles, seeking to avoid inducing agglomeration during the solidification process (pulsed mode), thereby providing specific information concerning the microstructural and mechanical properties of the composites (AMMCs). We applied a thermal treatment simulating an artificial aging treatment (as pure aluminum cannot be aged), analyzing the interaction of the zirconium oxide with the aluminum matrix, in order to improve the mechanical properties of the composite for possible industrial applications.

## 2. Materials and Methods

### 2.1. Composite Fabrication

**Figure 1:**
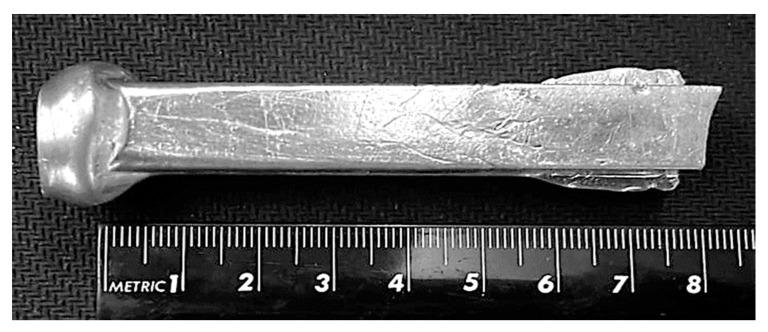
The as-cast sample of the MMC supplemented with ZrO_2_.

Composites were fabricated using a Felisa TE-M20D furnace (Guadalajara, Mexico). Pure aluminum (99.99%) was melted inside a stainless-steel crucible. In pulsed mode, 0.15, 0.25, and 0.50 vol.%. of zirconium dioxide (ZrO_2_-99%, Al-0.06, Fe-0.02%, Pb-0.02%) in liquid phase at 900 °C was added, with stirring simultaneously carried out at a constant speed of 250 rpm until a homogeneous mixture was obtained. Afterwards, the composite was cast in a copper mold; an image of the as-cast bar is presented in Figure 1.

The obtained samples corresponding to each addition were sectioned into coupons with dimensions of 1 × 1 × 1 cm. Sample designations and content are shown in Table 1.

### 2.2. Thermal Treatment

AMMC samples supplemented with different amounts of ZrO_2_ were heated at 450 °C and immediately cooled down using different media. Cooling rates were calculated according to the cooling media employed. The following values were obtained: 75.4 °C/s for the samples cooled in synthetic oil, 95.8 °C/s for the water-cooled samples, and 135.2 °C/s for the samples cooled in artificial brine. Then, the samples were thermally treated (simulating artificial aging) by heating them at 200 °C for 1 h and subsequently air-cooled.

### 2.3. Surface Preparation

To analyze the surface microstructures, the coupons were sanded using up to 600-grade sanding paper and polished with up to 0.03 µm of alumina. Immediately afterward, the samples were etched with Dix–Keller reagent (2.5 mL of HNO_3_, 1.5 mL of HCl, 1 mL of HF, and 95 mL of distilled water) for 20 s [23]. Microstructure images were obtained using a Leo 450 VP (Southampton, UK) Scanning Electron Microscope (SEM). Porosity analyses were performed using Image-Processing Analysis (IPA) equipment.

### 2.4. X-Ray Diffraction

Surface samples were prepared using sandpaper (up to 1200-grade) and, to avoid excessive alumina formation, immediately analyzed using a Bruker D2- PHASER X-ray diffractometer (Billerica, MA, USA), using a Cu-Kα X-ray tube, with λ = 0.15406 nm radiation and a scanning speed of 3°/min. The equipment’s internal software EVA, Version V3.1-2009, was used for phase determination.

### 2.5. Mechanical Testing

Hardness evaluations were carried out using a Leco 300 MT Microhardness Tester (St. Joseph, MI, USA), applying a load of 0.1 kgf and a holding time of 15 s, according to the ASTM: E384-22 standard [24]. Each measurement was carried out 10 times. Tribological evaluations were carried out using a conventional pin-on-disk TRB^3^ Anton Paar Tribometer (Graz, Austria) wear system, applying a constant load of 0.5 kg at a sliding distance of 4 km, with zero lubrication. The counterpart was a steel disk AISI-1045 (cold-rolled, low-carbon, and low-alloying), with an average hardness of 170 HVN. The ASTM G99-17 standard [25] was used as reference to perform the wear tests.

## 3. Results and Discussion

### 3.1. Microstructural Characterization

**Figure 2:**
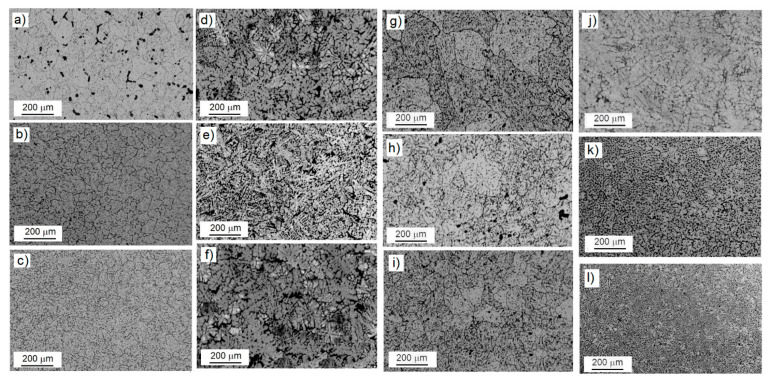
Microstructures of the samples supplemented with different amounts of ZrO_2_ and subjected to different cooling rates after heat treatment. M1: (**a**–**c**). M2: (**d**–**f**). M3: (**g**–**i**). M4: (**j**–**l**).

**Figure 3:**
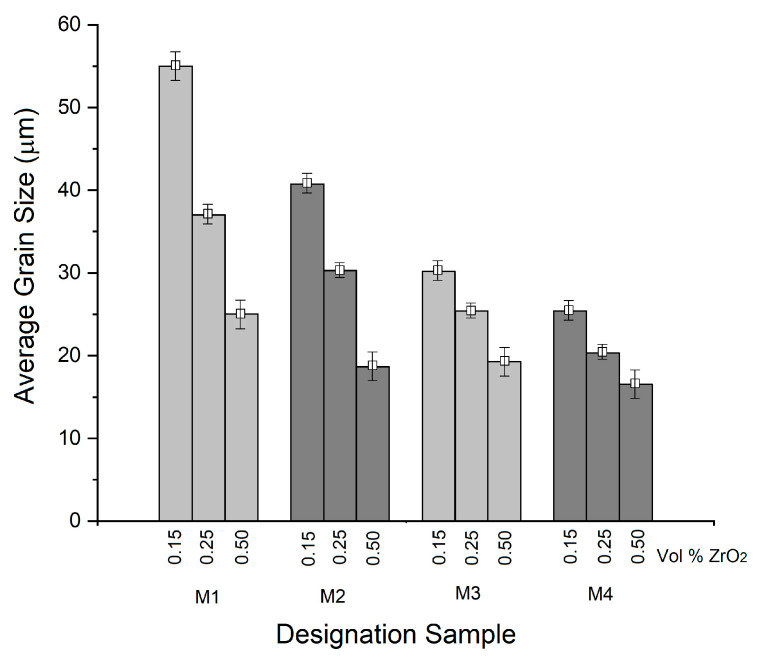
Grain size evaluation for the samples that were supplemented with ZrO_2_ and underwent different cooling rates.

These AMMCs reinforced with ZrO_2_ vol.% particles have a dense structure because of the lower degree of particle agglomeration (as confirmed through the following microstructural observations), which is mainly due to the stir-casting process. Figure 2 shows the microstructures obtained from samples M1 to M4, with 0.15, 0.25, and 0.50 vol.% of ZrO_2_ and different cooling rates (samples M2 to M4 underwent thermal treatment). Figure 2a–c correspond to the microstructures of sample M1 in the as-cast condition, indicating a porosity of approximately 1.872% of the area fraction (Figure 2a). Also, particles of ZrO_2_ are homogeneously distributed on the surface of the samples, with the average grain size being 55 µm, while for the samples with 0.25 and 0.50 vol.%, there is minimal porosity, and the average grain size diminishes to 25 µm. As pointed out by Reddy et al. [18], particles added during the melting–solidification process may act as nucleation points on which aluminum grains solidify, leading to a lack of porosity. In the case of sample M2 (Figure 2d–f), the presence of dendrites on the surfaces of the samples can be observed for the three different zirconia concentrations. This behavior can be attributed to the presence of particles (from ZrO_2_) along the grain boundaries that inhibit grain growth [19,20]. Figure 2g–i are images of sample M3 with different vol.% of ZrO_2_ added, showing a grain evolution consisting of the formation of columnar grains and, in a few zones, equiaxed grains. This effect is a product of the introduction of zirconium oxide inside the alloy matrix, leading to non-homogeneous grain growth due to oxide segregation towards the grain boundaries [18]. Figure 2j–l show the microstructures of sample M4, predominantly showing a globular α-Al-phase microstructure surrounded by uniformly distributed ZrO_2_ particles, with minimal porosity (1.284%) and refined intermetallic phases. Here, particles can be observed not only in the grain boundaries but also in the matrix of the composite. Figure 3 reveals the average grain sizes obtained for the samples. The most noticeably affected samples are samples M2 to M4, with grain sizes of 18.64, 19.26, and 16.56 µm, respectively, at a concentration of 0.50 vol.%. This variation in grain size suggests that ZrO_2_ particles play an important role in grain growth since the quantities in which they were added were sufficient to inhibit dislocation mobility. In other words, the increase in the reinforcement of ZrO_2_ vol.% contributes to a decrease in the grain size of Al via restricting grain growth; consequently, the grain boundaries serve as dislocation accumulation sites, leading to the formation of sub-grains and therefore small grains [18,19,20].

### 3.2. X-Ray Diffraction Analyses

**Figure 4:**
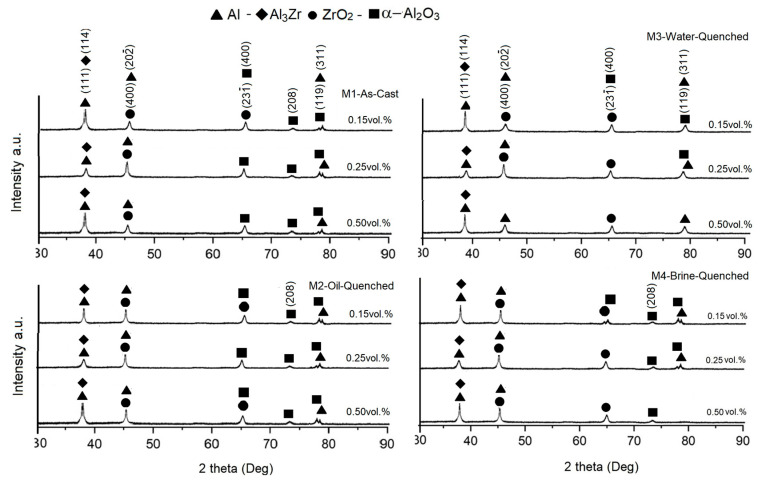
X-ray diffraction patterns of the AMMCs with different concentrations of reinforcement and different cooling rates.

The X-ray diffraction patterns for the AMMCs with different concentrations of reinforcement and different cooling rates are shown in Figure 4. The main peaks indexed for Al and ZrO_2_ were observed in all diffraction patterns at a Bragg angle of 2Ꝋ = 38, 45, and 65 degrees. The addition of reinforcement also led to the formation of compounds such as Al_2_O_3_ and Al_3_Zr owing to the cooling process the samples underwent after heat treatment. According to reports in different investigations related to the Al-Zr binary system [21,22], there are several stable and unstable compounds. Murray et al. [26] postulated that there are ten stable phases in the mentioned system: Zr_3_Al, Zr_2_Al, Zr_5_Al_3_, Zr_3_Al_2_, Zr_4_Al_3_, Zr_5_Al_4_, ZrAl, Zr_2_Al_3_, ZrAl_2_, and ZrAl_3_. In this study, although at a low intensity, a few of these compounds were detected in the XRD diffraction patterns of this Al-ZrO_2_ composite. After the samples were subjected to different cooling rates and heat-treated, the XRD patterns showed the formation of these same compounds (Al_3_Zr and Al_2_O_3_). The observed peaks, with relatively high intensities, correspond to the formation of Al_2_O_3_ in the system and are a product of an exothermic reaction between aluminum and the oxygen present in the atmosphere. The diffraction patterns of the heat-treated composites indicated the presence of other peaks at a relatively low intensity, indicating the formation of new phases. Thus, in these treated alloys, aluminum in the matrix reacts with oxygen present on the surface of the sample to create alumina; this is the main reaction that occurs in situ. Then, aluminum also reacts with the added ZrO_2_ powders to form Al_3_Zr plus alumina. Finally, the reaction between oxygen present in the atmosphere and Al_3_Zr occurs, creating alumina plus ZrO_2_, according to the following reactions [27]:

2 Al + 3/2 O_2_ → Al_2_O_3_ (1)

13 Al + 3 ZrO_2_ → 2 Al_2_O_3_ + 3 Al_3_Zr (2)

2 Al_3_Zr +13/2 O_2_ → 3 Al_2_O_3_ + 2 ZrO_2_ (3)

Equation (2) is the aluminothermic reaction between aluminum and zirconium dioxide. Lattice parameter variation was corroborated by determining the peak displacement in the Bragg angle. The diffraction patterns show peaks with higher intensities at 2Ꝋ—lower values (with ZrO_2_ addition and the treatment applied) compared to the peaks observed in the XRD patterns of the as-cast compound, where the main peak appears at an intensity of 2Ꝋ = 38 degrees, indicating a decreased Bragg angle, which indicates an increase in the lattice parameter of aluminum. This effect is a product of the incorporation of ZrO_2_ into the AMMCs, wherein ZrO_2_ interacts with the aluminum crystal structure (4.05 Å) when zirconium is dissociated and diffuses in a substitutional way, or oxygen diffuses via an interstitial mode; either of these two possibilities have an impact on lattice distortion, which may potentiate the mechanical behavior inhibiting dislocation mobility. The percentage change in the lattice parameter was calculated by taking into account the shift in the Al peaks in the Bragg angle. It was determined to be on the order of 5.58%, which would definitely generate residual stresses that promote dislocation motion.

### 3.3. Mechanical Behavior

#### 3.3.1. Hardness Evaluation

**Figure 5:**
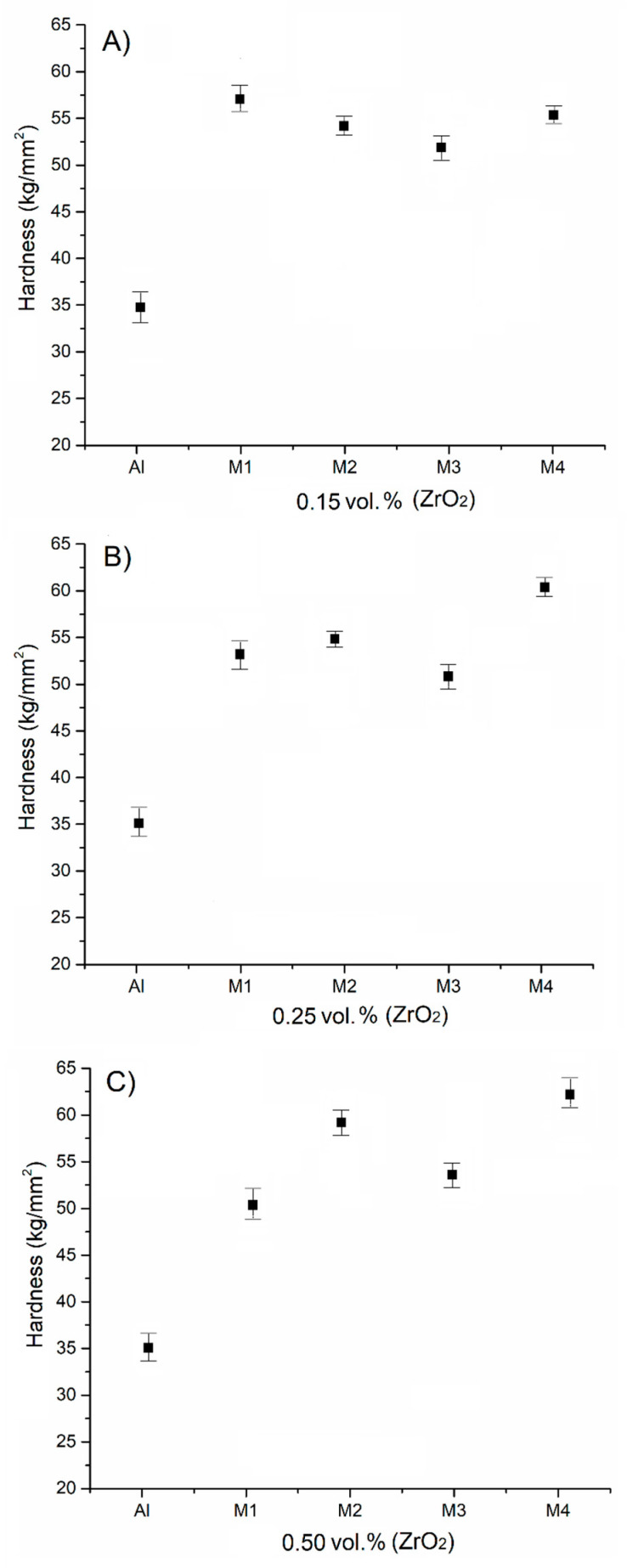
Plot of the hardness values of the AMMCs with different concentrations of reinforcement—(**A**) 0.15 vol.%, (**B**) 0.25 vol.%, and (**C**) 0.50 vol.%—each with different cooling rates, including the hardness value of the pure aluminum as comparison.

Due to the intrinsic low hardness of the aluminum matrix, the addition of ZrO_2_ particles as reinforcement was necessary in order to restrict plastic flow [27]; this was followed by an accelerated-cooling treatment to induce additional lattice distortion. In other words, the hardness of AMMCs is correlated with the degree to which the dislocation movement interacts with the reinforcement particles. Figure 5 shows a plot of the hardness values of samples M1 to M4 supplemented with ZrO_2_. Figure 5A is a plot of the hardness values for samples with 0.15 vol.% ZrO_2_, where it can be observed that sample M1 without thermal treatment (in the as-cast condition) shows a higher hardness value than samples M2 and M3, suggesting that lattice distortion occurred after the casting process and slightly affected the hardness values of the composite. However, although the hardness between samples M2 and M4 is similar, it is important to note that all the Al-ZrO_2_ composites with this composition presented a noticeable hardness increase of approximately 80% in comparison with pure aluminum. Figure 5B shows the hardness values for the samples with 25 vol.% of ZrO_2_. Evident, hardness increased approximately two times compared with the unreinforced sample (pure aluminum). However, the hardness values of the heat-treated samples increased slightly; hence, the aluminum matrix reinforced with medium amounts of ZrO_2_, in combination with the different cooling rates, exhibited an average improvement in hardness of 43% in comparison with the hardness of pure aluminum. The maximum hardnesses achieved at this concentration correspond to 55.2 HV and 60.1 HV for samples M3 and M4, respectively.

Figure 5C shows the hardness behavior of the samples with 0.50 vol.% of ZrO_2_, revealing that the treated samples have higher hardness values than the pure aluminum sample. Meanwhile, sample M4 showed an increase in hardness of 20% in comparison with samples M1, M2, and M3. Also, a hardness decrement in sample M3 can be observed, probably due to porosity below the surface. In contrast, hardness increased in sample M4 because of the increase in density and the distribution of ZrO_2_ particles. Moreover, ZrO_2_ particles also segregated throughout the grain boundaries, resulting in a hardness increase due to the inhibition of grain growth [28]. The cooling rate is a very important factor with respect to mechanical properties. This variation is a product of the microstructural modification of the material, where, depending on the quenching severity (e.g., medium, in this case), an ordered structure can be obtained when a slow cooling rate is applied, or a structure with a high percentage of residual stress and dislocation can be obtained when abrupt an cooling rate is applied.

The increase in the hardness of the AMMCs can be discussed in terms of a particle-hardening mechanism from a microstructural perspective: through the addition of ZrO_2_, as pointed out in previous paragraphs, it is possible for the displacement of crystal planes to inhibit dislocation movement [28], allowing the noticeable hardness increase observed to occur due to the different amounts of ZrO_2_ added, as ZrO_2_ is not totally present in the matrix as a mono-structured powder but instead forms small agglomerates, which segregate towards the grain boundaries, as shown in Figure 2.

#### 3.3.2. Wear Evaluation

**Figure 6:**
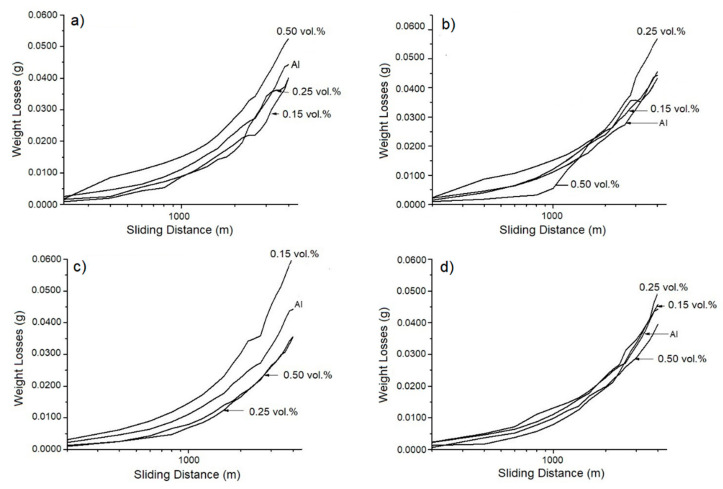
Plots of the wear evaluations of the AMMCs with different concentrations of ZrO_2_ and different cooling rates: (**a**) as-cast, (**b**) mineral oil, (**c**) water, and (**d**) synthetic brine.

Tribological analyses are important, mainly because they reveal the susceptibility of any materials to wear when they are in contact with other material during movement [19,21,29]. Figure 6a shows plots of weight loss against sliding distance for sample M1 in as-cast condition with different amounts of zirconium oxide added. The samples supplemented with 0.15 and 0.25 vol.% of zirconia exhibited constant wear behavior from the start of the test to its end. However, samples supplemented with higher amounts of zirconia are known to undergo considerable weight loss during tests owing to an abrasion mechanism due to the differences in hardness between aluminum and zirconia particles [30]. Therefore, these results can be attributed to the creation of an unstable abrasive layer with zirconium oxide and aluminum (from the matrix) during the wear process, which, due to contact and movement, is detached, thus leading to constant weight loss via an abrasive wear mechanism. Figure 6b shows the wear curves obtained from the worn samples of M2 cooled in mineral oil. Evidently, the sample with 0.50 vol.% presented minimal weight loss up to one thousand meters, showing a stable plateau after this stage, indicating that an oxide layer has formed between the surface sample and its counterpart. After this distance, the layer detached from the worn surface, leading to considerable weight loss. It is important to mention that during this period, several plateaus formed, with a cyclic tendency. In contrast, the sample supplemented with 0.25 vol.% of zinc exhibited constant weight loss during the test, indicating the creation, evolution, and depletion of an oxide layer [31,32]. For the sample with 0.15 vol.% reinforcement, at up to one thousand meters, the degree of weight loss was higher relative to the samples with a high zirconia content, indicating that zirconia particles significantly influence the wear process owing to the unstable oxide layer on the worn surface [30,31,33,34]. The weight loss values of the samples of M3 cooled in water are presented in Figure 6c. The three compositions of reinforcement added exhibited a similar behavior, mainly because the cooling rate related to brine temperature permits a homogeneous distribution of the zirconium oxide into the aluminum matrix [29,32]; it can also be observed that the sample with 0.15 vol.% of reinforcement lost weight immediately, unlike the samples with 0.25 vol.% and 0.50 vol.% of zirconia added, for which weight loss increased gradually. Figure 6d shows the wear plots for the samples of M4 cooled in synthetic brine, where it is noticeable that the sample with a low amount of zirconia added (0.15 vol.%) presented the most significant weight loss, while the samples with 0.25 and 0.50 vol.% of reinforcement exhibited moderate wear behavior.

Regarding the mechanism that governs the formation of the protective oxide layer against material wear, it is first important to note that, initially, the Al_2_O_3_ was formed spontaneously at room temperature. Subsequently, due to the friction generated by contact and the relative movement between the aluminum alloy and the steel counterpart, frictional energy is generated, which produces the heat necessary to form new oxides on the material and the added ZrO_2_, which is on the surface of the material, leading to an agglomeration of a mixture of oxides with the detached material, namely, an oxide layer (the plateau in the wear curve), which, with the passage of time and/or an increase in sliding distance, becomes detached, leading to the weight loss observed in the wear curves, characterized by a slope in the curve.

#### 3.3.3. Wear Factor Evaluation

**Figure 7:**
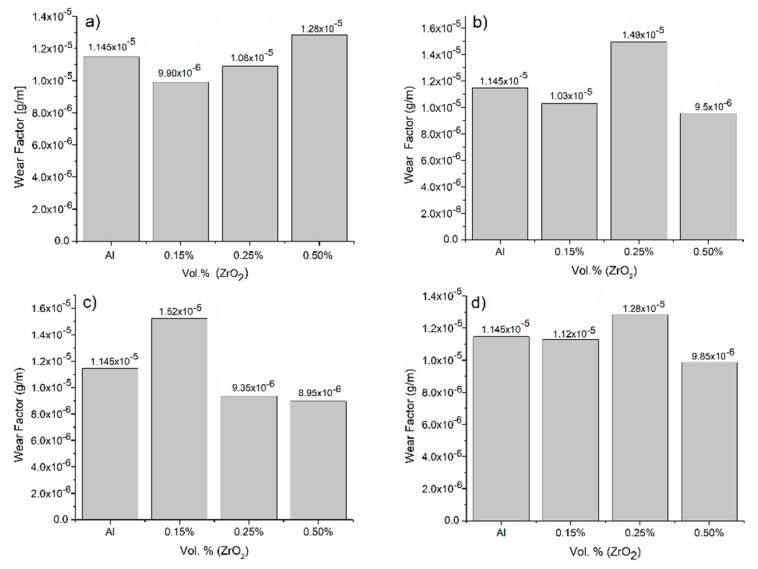
Plots of the wear factor obtained from the wear plots for the different AMMCs supplemented with different amounts of zirconia and subjected to different cooling conditions: (**a**) as-cast condition, (**b**) mineral oil, (**c**) water, and (**d**) brine medium.

Because the wear factor is a parameter that directly correlates weight loss with sliding distance, it can reveal the magnitude of wear experienced the detached material during the wear process, no matter which type of mechanism is involved [32,35]. Thus, Figure 7a shows a plot of the wear factor for the worn samples in the as-cast condition, where it is evident that the sample supplemented with 0.50 vol.% of zirconia is the one with a greater susceptibility to weight loss. This result is not surprising since ZrO_2_ particles, in combination with the aluminum matrix, create an unstable oxide layer with poor cohesion with respect to the surface of the worn sample; this is because the energy required to create internal strong bonds is not available [29,32,35,36,37]. Hence, detached oxides mixing with aluminum from the matrix is highly likely. For the samples cooled in mineral oil and water (Figure 7b and Figure 7c, respectively), the samples with 0.50 vol.% of reinforcement showed the best resistance to weight loss, with a 20 percent increase in their wear resistance under this applied load. In contrast, Figure 7d presents the values of the wear factor for the samples cooled in a brine medium, where it is noticeable that the samples supplemented with 0.25 and 0.50 vol.% of ZrO_2_ present the lowest wear factor values in comparison with the aluminum sample. In these evaluations, the operating mechanism is predominantly oxidative–adhesive: The necessary temperature for oxide formation is easily reached due to the contact between the sample and its counterpart via relative movement during the test (enough frictional energy is produced) [35], so the oxide layer produced adheres strongly to the two surfaces until saturation is achieved, thereby generating a lubricant layer. Then, the oxide layer is ejected from the system, once again initializing a new oxidation cycle. The temperature at which oxide formation occurs due to frictional heating in the aluminum alloys and at this applied load is approximately 125 °C, although this temperature varies according to the delamination produced. Furthermore, it is important to consider that an increase in weight loss can also be directly related to surface softening due to heating via friction. This phenomenon occurs a few microns below the worn surface, where surface contact between the worn sample and its counterpart generate enough heat to modify the structure of the alloy, with the surface sample being more affected than the surface of the counterpart because of the difference in volume; this shows the importance of selecting the right method for melting the alloy (such as the stir-casting method) [37,38] in order to obtain samples with homogeneous particle dispersion.

#### 3.3.4. Wear Surface Analyses

**Figure 8:**
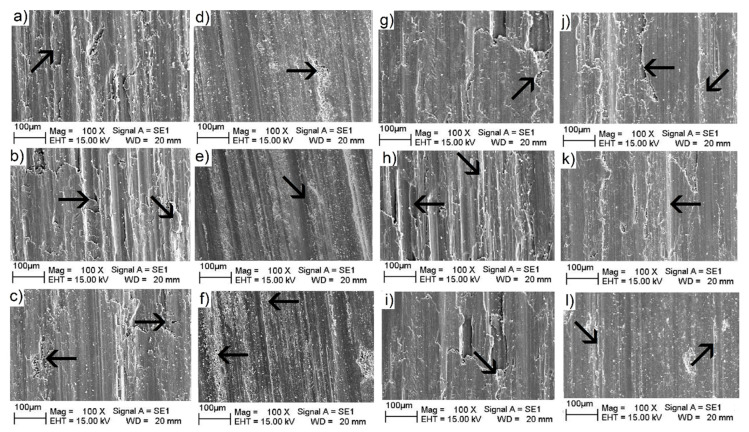
Surfaces images of the worn AMMCs indicating the zones of oxide formation and the grooves formed by abrasive particles (bright zones indicate oxide agglomeration). Arrows indicate zones with high oxide concentrations.

Figure 8a–c show worn-surface images of the samples with 0.15 vol.%, 0.25 vol.%, and 0.50 vol.% of zirconium oxide added, respectively, where a combination of wear scratches and oxide agglomerations can be observed along the worn surface. Hence, as shown in Figure 8a, for the as-cast sample, the scratches generated during the wear process are deeper, which indicates that abrasive particles scratch the surface easily due to the low hardness of this sample, which, in fact, possesses the lowest surface hardness [29]. In Figure 8b,c, it can be observed that some oxide delamination zones (marked by arrows) were produced, presumably due to the heating reaction between the aluminum and zirconia particles and oxygen in the atmosphere, creating unstable oxide agglomerates with low superficial adhesion on the worn surface [32].

Figure 8d–f present images of the worn surfaces of the oil-quenched samples supplemented with 0.15 vol.%, 0.25 vol.%, and 0.50 vol.% of ZrO_2_, respectively. Evidently, wear tracks are homogeneously distributed over the worn surfaces for all the different concentrations of ZrO_2_. Arrows in the images show the different zones where oxide accumulation occurred. Importantly, the sample with the higher zirconia content (Figure 8f) presents deeper wear tracks (corresponding to the arrow in the left zone), indicating a depletion of the oxide layer [39,40] relative to the samples with lower zirconia content. In this case, the operating wear mechanism turned out to be the oxidative type [29]. Figure 8g–i present worn-surface images of the water-quenched samples with 0.15 vol.%, 0.25 vol.%, and 0.50 vol.% of ZrO_2_, respectively, where it can be observed that the wear tracks for the three different compositions present similar patterns, with a moderate degree of surface oxide formation. Hence, the associated wear mechanism for this set of samples is a combination of oxidative and abrasive modes [39,41]. For the samples cooled down in a brine environment (Figure 8j–l, with 0.15 vol.%, 0.25 vol.%, and 0.50 vol.% of ZrO_2_, respectively), the sample with 0.50% zirconia (Figure 8l) presents the main concentration of superficial wear tracks, indicating high hardness over the surface sample. Also, there are very few visible zones on the three surface samples showing evidence of oxide agglomeration due to frictional heating (marked by arrows), which is the main operating mechanism of the oxidative–abrasive type [29]. It has been reported that after thermal treatment of composites, an increase in toughening occurs, with a reduction in cracking tendency [42,43].

## 4. Conclusions

AMMCs reinforced with different amounts of ZrO_2_ (in vol.%) were produced using the stir-casting method. The samples were cooled at different cooling rates (using different cooling media) and then subjected to a thermal treatment.

- The samples with low amounts of ZrO_2_ added showed a refined dendritic microstructure, while elevated amounts of reinforcement and accelerated cooling rates led to equiaxed grains. Grain size was directly affected by the amount of reinforcement added to the MMCs.
- An evaluation of the hardness of the samples reinforced with 0.50 vol.% of ZrO_2_ and subjected to thermal treatment that were cooled in synthetic brine showed they exhibited the greatest increase in their values, reaching approximately 30 percent (60 kg/mm^2^) higher in comparison with the sample without reinforcement.
- Tribological evaluations showed that the samples supplemented with 0.50 vol.% of zirconia and oil-quenched presented the lowest weight loss and therefore elevated wear resistance. Two wear mechanisms were identified: oxidative–adhesive for low concentrations and abrasive for higher zirconia concentrations.

The distortion of the lattice of the aluminum structure due to zirconia addition and hardening due to particle addition (ZrO_2_) are the proposed strengthening mechanisms in this case (resulting in dislocation mobility inhibition). Transmission electron microscopy studies will be performed to obtain more-detailed information on the hardening mechanisms of the composite produced by adding ZrO_2_, thus filling in some information missing in this research.

## Figures and Tables

**Table 1:** AMMC composition and nomenclature of samples obtained under different cooling rate conditions classified according to severity from M1 to M4.

Sample Designation	Heat Treatment	Designation	ZrO_2_ Additions in [vol.%]	Porosity (%)
M1	As-cast	(a)	0.15	1.872
		(b)	0.25	1.484
		(c)	0.50	1.732
M2	Oil quenched + thermal treatment	(d)	0.15	3.323
		(e)	0.25	**2.567**
		(f)	0.50	3.425
M3	Water quenched + thermal treatment	(g)	0.15	2.220
		(h)	0.25	1.914
		(i)	0.50	2.362
M4	Brine quenched + thermal treatment	(j)	0.15	3.451
		(k)	0.25	2.321
		(l)	0.50	1.284

## Data Availability

The original contributions presented in this study are included in the article. Further inquiries can be directed to the corresponding author.
